# Beyond Single Chains: Benchmarking Macromolecular Complex Prediction Methods With the Continuous Automated Model EvaluatiOn (CAMEO)

**DOI:** 10.1002/prot.70060

**Published:** 2025-09-28

**Authors:** Xavier Robin, Peter Škrinjar, Andrew M. Waterhouse, Gabriel Studer, Gerardo Tauriello, Janani Durairaj, Torsten Schwede

**Affiliations:** ^1^ Biozentrum, University of Basel Basel Switzerland; ^2^ SIB Swiss Institute of Bioinformatics Basel Switzerland

**Keywords:** 3D structure prediction, CASP16, macromolecular complexes, protein structure, protein‐ligand complexes

## Abstract

Independent, blind assessment of structure prediction methods is essential for establishing state‐of‐the‐art performance, identifying limitations, and guiding future developments. The Continuous Automated Model EvaluatiOn (CAMEO) platform provides weekly, automated benchmarking of structure prediction servers, complementing the biennial Critical Assessment of Structure Prediction (CASP) experiments.

## 1 Introduction

Far from being the end of three‐dimensional (3D) biomolecular structure prediction research as some feared initially, the release of AlphaFold 2 in 2020 [1] marked the beginning of a new era in 3D modeling, where the focus of the community shifted away from single‐chain protein structure prediction toward the prediction of macromolecular complexes and assemblies. In response to this evolution, new categories such as RNA structure prediction, protein‐ligand complexes, or the estimation of model accuracy for oligomeric targets were introduced in CASP15 [2]. The Critical Assessment of Structure Prediction (CASP) is organized every 2 years and brings together experts to independently assess methods using a few hundred prediction targets in a double‐blind setting. It culminates in a meeting where assessors and researchers compare and discuss the prediction methods.

As the field of structure prediction continues to evolve, the need for systematic and unbiased benchmarking remains fundamental. However, achieving fair comparisons is challenging due to variability in datasets, evaluation metrics, and methodological assumptions. To support the development of automated server methods, more frequent benchmarking on larger datasets between CASP seasons is essential for testing hypotheses and accelerating development cycles. Since 2012, the Continuous Automated Model EvaluatiOn (CAMEO) platform [3, 4, 5, 6] has filled this gap by providing weekly, independent, blind assessments of structure prediction servers. Leveraging the two‐phase update cycle of the Protein Data Bank (PDB) [7], CAMEO selects targets shortly before their experimental structures are released and distributes them to participating servers. Once the reference structures become available, predictions are evaluated using a suite of community‐established metrics that capture various aspects of structural accuracy.

To ensure a fair comparison, CAMEO evaluates all servers simultaneously. This guarantees that each method has access to identical background information, including sequence and template data. All results (including predictions, reference structures, and evaluation scores) are publicly available, allowing method developers to track performance and use the data for training and validation. Developers can register their methods as either public or development servers, allowing anonymous benchmarking during early‐stage testing. To support reproducibility and historical comparisons, CAMEO encourages participants to maintain previous versions of their servers alongside new releases.

This manuscript presents recent developments in CAMEO that reflect the field's growing emphasis on macromolecular complexes. CAMEO has expanded its scope to include entire complexes composed of proteins, nucleic acids, and small‐molecule ligands. Determining the novelty of such complexes remains an open challenge. Although methods based on existing structural information have been proposed [8, 9, 10], this data is unavailable to CAMEO at the time of target submission. To address this, CAMEO employs automated procedures based on sequence identity and clustering to detect novelty and classify targets. In parallel, new baseline methods have been introduced to benchmark performance across a broader range of prediction tasks. The CAMEO website has also been redesigned to better visualize server performance across diverse target types. All benchmarking results are accessible at cameo3d.org.

## 2 Results

### 2.1 Target Selection

CAMEO performs weekly, independent, blind benchmarking of registered prediction servers by leveraging the two‐phase update procedure of the Protein Data Bank (PDB) [7]. Each Saturday (Phase 1), a list of new PDB entries is downloaded. A CAMEO target is defined as a complete macromolecular complex, including all polymer (nucleic or amino acid) and non‐polymer (ligand) entities.

To identify meaningful modeling challenges, CAMEO filters and clusters sequences to avoid redundancy, then aggregates clustering results at the complex level. These clustered entries are compared to the latest public PDB release to assess novelty. Since no standard method exists to determine the novelty of a complex based solely on sequence, CAMEO uses a combination of sequence similarity and aggregation rules to classify targets as “easy,” “medium,” or “hard.” Easy targets containing novel ligand contexts are further labeled as “ligand” targets. A result of this classification is that all sequences in a target may have templates with high sequence identity in the PDB; however, if they have not been observed together in the same complex, we deem the complex interesting and send it to CAMEO participants.

### 2.2 Benchmarking Challenges

CAMEO participants can subscribe to specific target categories (“ligand,” “medium,” or “hard”) based on their server capabilities. They may also choose to receive only protein, DNA, or RNA sequences, or opt in to receive full non‐canonical sequences to tackle non‐standard compounds. To ease the transition for former CAMEO participants, servers that can only model a single sequence at a time can receive entries containing a single sequence only.

In the context of macromolecular complexes, stoichiometry refers to the number and identity of each molecular component (such as protein, RNA, DNA chains, and small‐molecule ligands) present in the biologically relevant assembly. A key challenge in CAMEO is that this information is not available in the PDB pre‐release, meaning prediction methods must infer the full composition of the complex without prior knowledge. Methods that correctly model complex composition are rewarded through stoichiometry‐aware metrics (e.g., LDDT, iLDDT), which penalize missing chains. To support fair interpretation of results, CAMEO also provides stoichiometry‐free variants (mapped LDDT and mapped iLDDT), which evaluate only the correctly predicted chains. This dual scoring approach allows users to distinguish between errors in structural accuracy and errors in complex composition, while still encouraging the development of methods that can model complete assemblies.

Participants must submit their prediction of full atomic models of the complexes by email before the end of the prediction window on Wednesdays at 00:00 UTC, when newly released entries are made available by the PDB (phase 2 of the weekly PDB update). CAMEO supports the same prediction formats as CASP16, ensuring compatibility with established workflows.

### 2.3 Comparison to Reference Baselines

To facilitate the interpretation of benchmarking results and provide context for evaluating the performance of prediction methods, CAMEO includes several reference baselines. For ligand‐containing targets, three baseline workflows are employed. Two of these are based on an open‐source pipeline that combines SWISS‐MODEL [11] for generating initial protein complex models with physics‐based AutoDock Vina [12] for ligand docking. These two variants differ in their scoring functions, using either the vina or the autodock4 [13] scoring schemes, and are representative of typical open‐source docking workflows. A third baseline integrates SWISS‐MODEL with physics‐based Schrödinger Glide, representing a commercial docking solution. None of these baselines incorporate a pocket prediction step, resulting in conservative performance estimates. Additionally, they naively select the top‐ranked model from SWISS‐MODEL, which is often based on structures from the AlphaFold database. As shown in our previous work [14], physics‐based methods consistently fail to dock ligands into AlphaFold models. Consequently, these workflows are intended to serve as lower‐bound references, against which improvements by more advanced methods should be readily observable.

In addition to these ligand‐specific baselines, AlphaFold 3 [15] (version 3.0.1) is used to generate models for all targets, including those containing nucleic acid sequences. This provides a consistent and comprehensive benchmark across all target types. AlphaFold 3 predictions are generated assuming a single copy of each chain, as stoichiometry information is not available at the time of target submission. Together, these baselines establish a robust framework for comparative evaluation, enabling the identification of strengths and limitations in emerging prediction methods.

### 2.4 New Website

**FIGURE 1:**
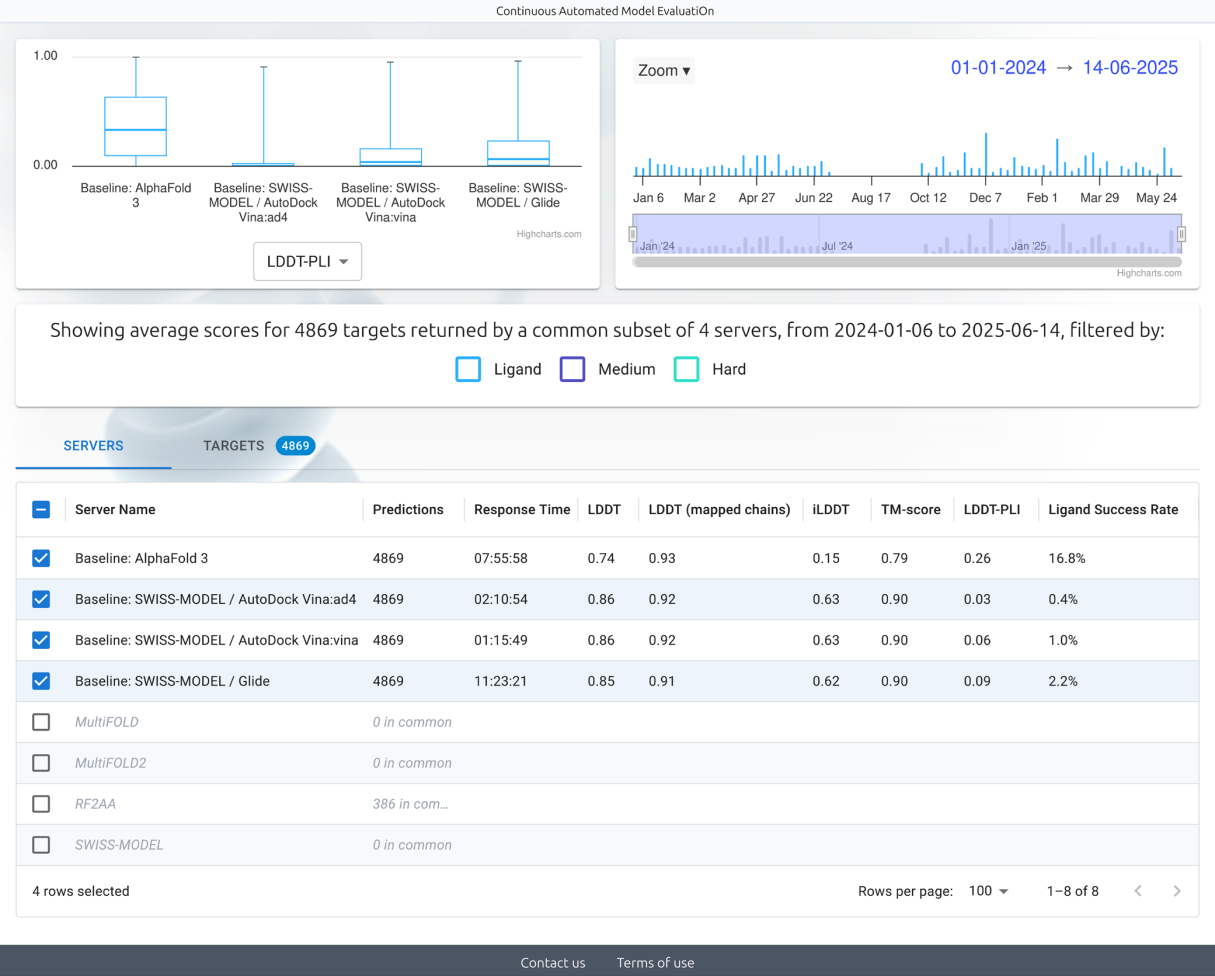
Screenshot of the results page. A selection of four baseline servers for protein‐ligand complex prediction over the 76‐week period from January 6, 2024 to November 30, 2024 analyzed in this manuscript.

In order to display benchmarking results for the new challenges, we redesigned the CAMEO web server. Figure 1 shows a screenshot of the main results page.

#### 2.4.1 Common Subset Comparisons

Depending on their subscriptions, servers receive disjoint sets of targets. In addition, they may not have built models for all the received targets. Therefore, simple comparison of averages over all targets over a given timeframe is meaningless. For that reason, only the total number of predictions is displayed by default, and we provide tools to compare servers based on common subsets of targets. Selecting a server in the list will automatically display the results for all the targets of the timeframe predicted by that server and list how many targets are overlapping with other servers. Selecting additional servers further restricts the common subset and adjusts the results for all servers. Users should only perform comparisons if the common subset of overlapping targets is sufficient to provide meaningful statistics.

CAMEO displays a selection of scores measuring different aspects of protein structure prediction accuracy, and doesn't establish a single unique ranking between the methods. The focus of CAMEO has always been on all‐atom scores to capture the ability of participants to accurately model proteins, including biologically relevant protein side chain conformations. In addition, as CAMEO is a fully automated workflow without human intervention, we have been focusing on superposition‐free scores, which alleviate the need to manually split proteins into evaluation units [6, 16, 17]. Score computation, including chain and ligand mapping, is performed with OpenStructure [18]. By default, all the scores penalize missing chains and ligands upon wrong or lack of stoichiometry predictions. The LDDT score (Local Distance Difference Test) [18, 19] is an all‐atom, superposition‐independent score that evaluates the local accuracy of protein or nucleic acid predictions by reporting the fraction of correctly predicted inter‐atomic distances in a model at different threshold levels. LDDT penalizes missing residues and chains in the model and applies a filter for stereochemical violations and steric clashes. In addition, we display the LDDT (mapped chain) score, which alleviates the penalty for missing chains. The LDDT is typically dominated by intra‐chain residues.

The iLDDT (Interface LDDT) [18] is a variation of LDDT that reports only inter‐chain contacts in order to assess the accuracy of polymer‐polymer interfaces. Due to the relatively small distance difference thresholds of LDDT, iLDDT is very sensitive to even small errors in interface predictions. This sensitivity should capture even small improvements in high‐accuracy interface prediction methods but will fail to distinguish moderately to completely wrong interfaces. The TM‐score [20] is a backbone‐only score for polymer predictions dependent on a global superposition to assess the overall accuracy of a complex. It mitigates the effect of outlier regions by focusing on maximizing the alignment of correctly predicted regions, limiting the influence of erroneous regions by treating them as outliers. The TM‐score contains a scaling factor d_0_ in order to be independent of the protein length. As a result, for large oligomeric complexes, local accuracy can be fairly low even with a high TM‐score.

In addition to scores for polymer chains, we provide scores to assess the accuracy of ligand poses. The LDDT Protein‐Ligand Interface (LDDT‐PLI) [18, 21] is a variation of LDDT that assesses the accuracy of polymer‐ligand contacts. Finally, successes are defined as ligand predictions with an RMSD < 2 Å after superposition of the binding site and symmetry correction (BiSyRMSD) [18, 21].

#### 2.4.2 Target Details

**FIGURE 2:**
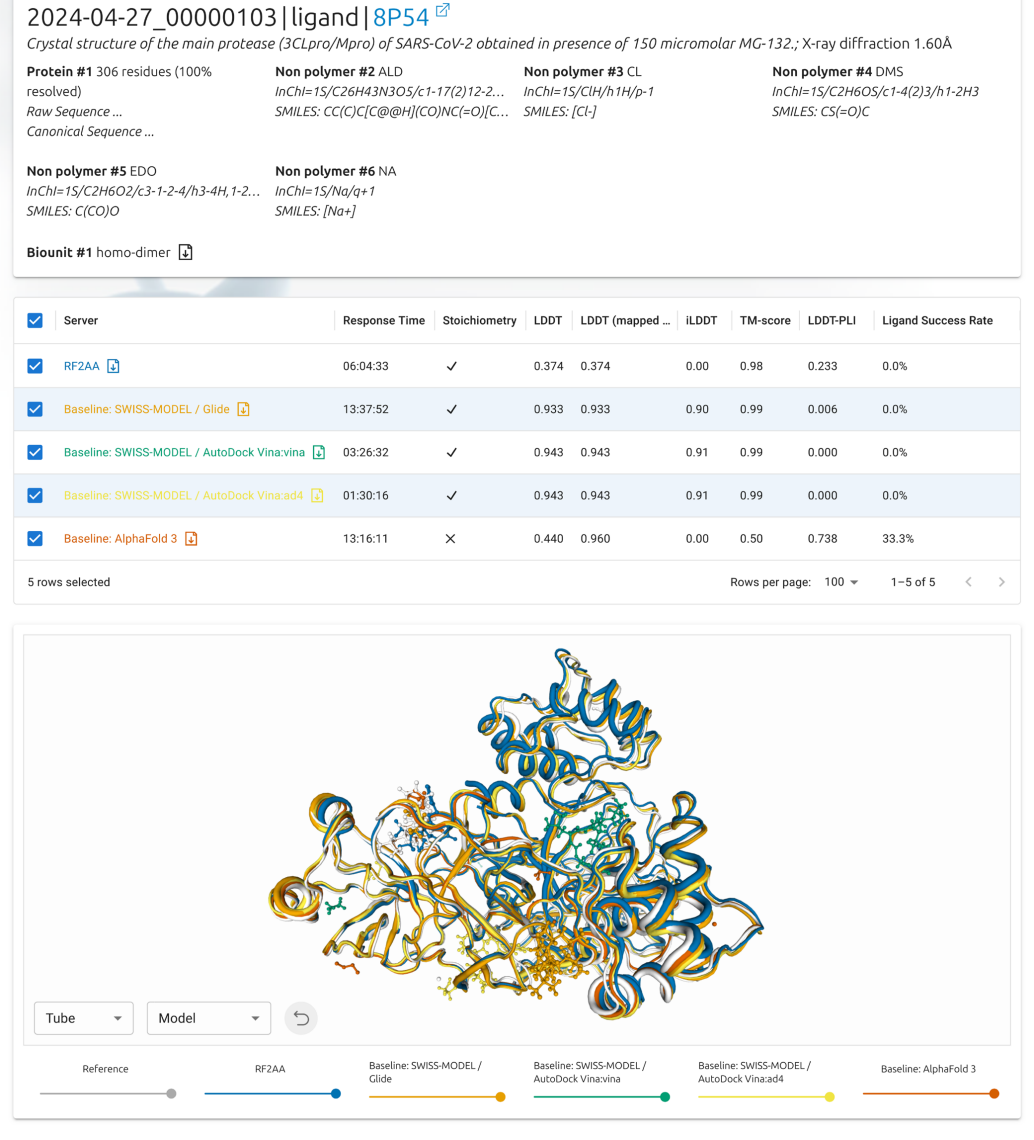
Target results page. Target 2024‐04‐27_00000103 and five models were selected and are displayed in colors in the 3D viewer.

The target details page (Figure 2) shows detailed information about a single target and the predictions (Model 1). Below general information about the target, the page lists all polymer (including sequence length and coverage) and non‐polymer entities, as well as information about the assembly (number and oligomeric state). The servers table shows the Model 1 predictions received from each server, as well as information about response time, oligomeric state, and scores. Ligand scores are aggregated with weighted means, as described in Section 3. Upon selection of the server, the 3D models are displayed in the 3D viewer below. By default, the models are color‐coded by server, matching the servers table. A drop‐down box allows users to switch to per‐residue LDDT scores.

#### 2.4.3 Downloads

Data downloads are available for various time frames. They contain target and prediction data, results for all models, as well as additional scores not shown on the web interface.

These downloads also allow users to perform a posteriori benchmarking of methods not included in CAMEO.

### 2.5 Data Analysis

For this study, we analyzed the performance of baseline predictors and CAMEO participants over a 76‐week period (January 6, 2024, to June 14, 2025) on two major challenges: protein‐ligand and protein–protein complex prediction. Out of 23 629 new PDB entries, 12 205 were selected as interesting targets: 2106 “medium,” 3266 “hard,” and 6833 “ligand.” Table 1 summarizes the molecular entity types across these categories.

**TABLE 1:** breakdown of molecular entities in CAMEO target by class and by entity type.

Target classification	Hard	Medium	Easy + ligand
Entity type			
RNA	448	3	27
DNA	1289	12	115
Protein	7609	3236	8125
Non polymer	3380	2765	18 015

#### 2.5.1 Protein‐Ligand Complex Prediction

**FIGURE 3:**
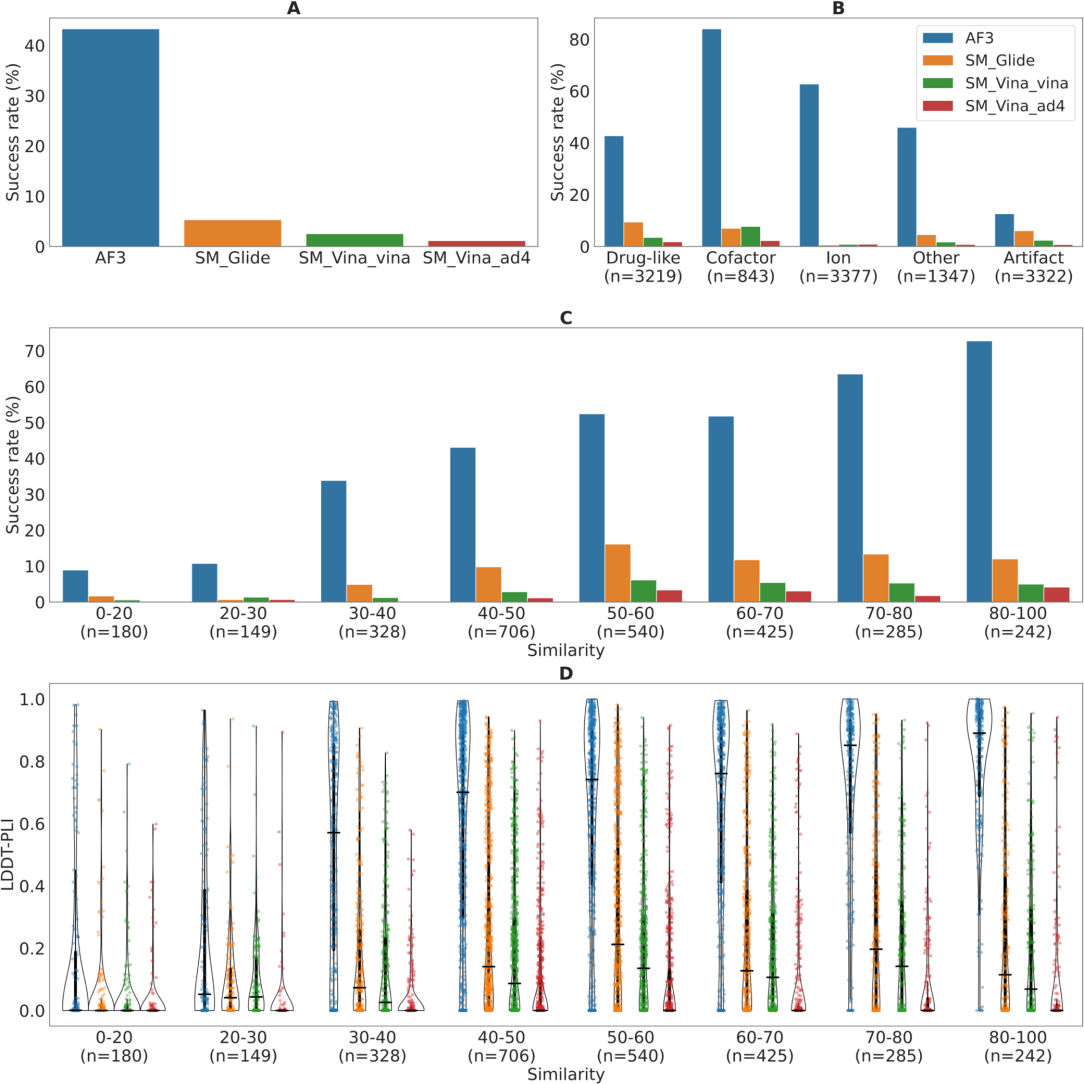
Baseline predictors for protein‐ligand complex prediction. The success rate, defined as the percentage of ligand entities with < 2 Å RMSD, across four baseline predictors for (A) all ligand predictions, (B) across different ligand categories, and (C) across different ligand similarity bins for the ligands in the drug‐like category. (D) The distribution of LDDT‐PLI values for four baseline predictors across different ligand similarity bins for the ligands in the drug‐like category. Baseline predictors: AlphaFold 3 (AF3, blue), SWISS‐MODEL homology modeling and ligand docking with Schrödinger Glide (SM_Glide, orange), Autodock Vina with vina scoring (SM_Vina_vina, green) and autodock4 scoring (SM_Vina_ad4, red). Similarity score could not be calculated for 364 drug‐like entities, therefore we excluded these from the difficulty analysis.

Using PLINDER [9] annotations and Runs N′ Poses [8] similarity definitions, we analyzed 6833 ligand targets, of which 6441 mapped to PLINDER, yielding 17 322 ligands. After excluding 392 non‐mapped targets (710 ligands) with metal complexes or obsoleted entries, ligands were categorized into five groups: “drug‐like” ligands, which satisfy Lipinski's Rule of Five and appear in fewer than 100 PDB entries (*n* = 4141); “cofactors” (*n* = 1493); “ions” (*n* = 4693); and “artifacts” (*n* = 4804), such as buffer molecules or precipitants from crystallization. The remaining 2191 ligands that do not fall into any of the four categories were labeled as “others,” primarily comprising metabolites, sugars, and lipids. We collected the results from baseline servers, which predicted 4864 targets in the common subset (12 108 ligands), and we analyzed the results of the top‐ranked models for each target (Figure 3). Here, we are taking the best‐scored pose per ligand entity; therefore, we are not penalizing for not predicting multiple copies of the same entity.

The overall analysis across all the entities shows significantly better performance of AlphaFold 3 compared to the other baseline predictors (Figure 3A). When examining individual categories, while AlphaFold 3 consistently outperforms other methods across most ligand categories, it shows reduced accuracy for artifacts (Figure 3B). Interestingly, its best performance is observed in predicting cofactor poses, likely due to the abundance of cofactor data available in the PDB, which was also shown in other studies [8]. Similar observations are seen when analyzing the predictions of another deep‐learning method in CAMEO, RoseTTAFold All‐Atom [22], although on a smaller ligand subset (Figure S1). Although the performance of AlphaFold on the drug‐like set is superior to other methods, the success rate of around 40% is significantly lower than reported on the PoseBusters dataset [15, 23].

Furthermore, we assessed the performance of baseline predictors across various levels of prediction difficulty for the ligands in the drug‐like category (Figure 3C). Prediction difficulty is defined based on the similarity to experimental structures in the PDB released before September 30, 2021, which corresponds to structures available as training data for AlphaFold 3, with lower similarity indicating higher difficulty, as detailed in Runs N′ Poses [8]. Our analysis shows that although AlphaFold 3 outperforms the other baseline predictors across all difficulty levels, its performance remains strongly correlated with the similarity to its training set. Interestingly, we also found that the performance of template and physics‐based methods correlates with the similarity to the AlphaFold 3 training set. This suggests that even when a protein target is considered “easy,” which is the case for this CAMEO category, challenges arise if the ligand binds to an alternative protein pocket or the protein binding pocket adopts a distinct conformation not previously seen in the PDB. A similar trend was observed in our analysis of the accuracy of protein‐ligand interaction prediction using LDDT‐PLI (Figure 3D).

This analysis demonstrates that while AlphaFold 3 consistently outperforms traditional docking baselines across most ligand categories, its performance remains closely tied to the availability of similar structures in its training data, particularly for drug‐like ligands. This highlights the importance of CAMEO's continuous and blind benchmarking framework, which exposes limitations in generalization and helps identify cases where current methods struggle with novel ligand contexts or alternative binding site conformations. By systematically capturing such edge cases, CAMEO provides a valuable resource for guiding the development of more robust and generalizable ligand modeling approaches.

#### 2.5.2 Protein–Protein Complex Prediction

**FIGURE 4:**
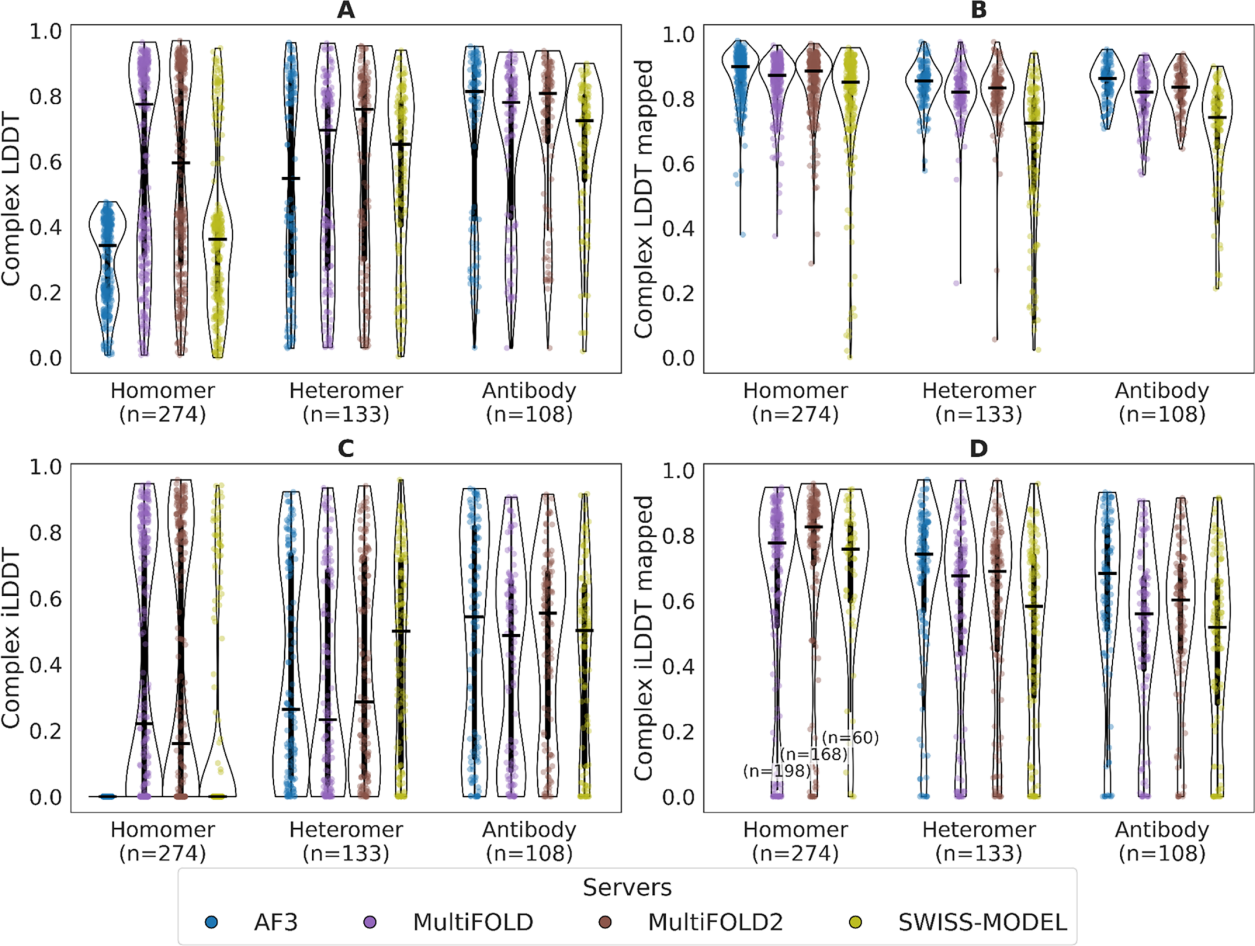
Protein–protein complex prediction comparison. The accuracy of protein–protein complex prediction of AlphaFold 3 (AF3, blue), MultiFOLD (purple), MultiFOLD2 (brown), and SWISS‐MODEL (yellow). Performance of servers is evaluated with (A) complex LDDT, (B) mapped complex LDDT, (C) complex iLDDT, and (D) mapped complex iLDDT.

We analyzed the performance of baseline predictors and participants on different categories of protein–protein complexes. There were 4482 targets in the hard and medium category when excluding the targets with DNA and RNA entities. For this analysis, we also excluded 1080 monomeric targets and classified the remaining 3402 targets into three categories: antibodies, which include targets that could be mapped to the Structural Antibody Database (SAbDab) [24] (*n* = 866); homomers (*n* = 1313); and heteromers, which are not in the antibody category (*n* = 1223). We compared the results of top‐ranked models from the AlphaFold 3 baseline server with three CAMEO participants (MultiFOLD [25], MultiFOLD2 [26], and SWISS‐MODEL [11]) on a common subset of 516 targets (Figure 4). As mentioned in the Challenges section, there is no information about the stoichiometry of the complex in the PDB pre‐release; therefore, methods which don't predict the stoichiometry are penalized by some scores. To address this, our analysis employs both “stoichiometry‐aware” metrics (LDDT and iLDDT, Figure 4A,C) and their “stoichiometry‐free” versions (mapped LDDT and mapped iLDDT, Figure 4B,D); see Section 3 for details.

The analysis of the overall structure prediction of the complexes with LDDT shows that MultiFOLD and MultiFOLD2 outperform SWISS‐MODEL and AlphaFold 3 in the homomer category, largely due to their capability to predict complex stoichiometry (Figure 4A). Even though SWISS‐MODEL can use stoichiometry information from the PDB templates, it appears that it mostly selects monomeric AFDB models for homology modeling, which shows in well modeled individual entities on the mapped LDDT for this category (Figure 4B). Similar observations are seen in the heteromer category, where MultiFOLD and MultiFOLD2 still outperform SWISS‐MODEL and AlphaFold 3, although both of them have slightly better performance than in the homomer category, due to the heteromers with only one protein chain per entity (62 out of 133 targets with such cases). In the antibody category this is even more obvious, where 75 out of 108 targets have only one protein chain per entity. As a result, AlphaFold 3 achieves similar performance as MultiFOLD2 (both median LDDT = 0.81) and slightly outperforms MultiFOLD (median LDDT = 0.78) within this category.

Similar trends of server performances are seen in the analysis of the interface predictions with iLDDT (Figure 4C,D). Monomeric predictions do not have a defined mapped iLDDT value in the homomer category (Figure 4D). As a result, there are no data points for AlphaFold 3 and fewer data points for SWISS‐MODEL (*n* = 60) than for MultiFOLD (*n* = 198) and MultiFOLD2 (*n* = 168). In general, we observed that interfaces are modeled less accurately than the overall structures (Figure 4A,C), even when we don't take stoichiometry into account (Figure 4B,D).

This analysis demonstrates that protein–protein complex prediction methods capable of modeling stoichiometry, such as MultiFOLD and MultiFOLD2, achieve higher accuracy in homomeric and heteromeric assemblies compared to baselines like AlphaFold 3. The latter performs best in antibody‐related targets, which typically involve a single binding interface. These findings highlight the importance of evaluating not only structural accuracy but also the completeness of predicted assemblies. By incorporating stoichiometry‐aware and stoichiometry‐free metrics, CAMEO enables a nuanced assessment of prediction methods and supports the development of tools that can handle the full complexity of macromolecular assemblies.

## 3 Methods

### 3.1 Target Classification

The initial CAMEO target set encompasses every newly released entry in the PDB [7]. The pre‐release data is downloaded on Saturday from the wwPDB website. It contains the non‐canonical sequence of every polymer entity (proteins, RNA, or DNA) as well as the Chemical Component ID [27], SMILES, and InChI code of every non‐polymer (small‐molecule ligand) entity, except oligosaccharides. A CAMEO target is an entire macromolecular complex (entry) from the PDB pre‐release, including all its entities. Interesting modeling targets are selected weekly from this set. Canonical sequences are derived from non‐canonical sequences using the PDB Chemical Component Dictionary.

In the current setup, targets are excluded if any of their polymer sequences contain modified residues that don't have a parent amino or nucleic acid in the PDB Chemical Component Dictionary, are a mix of nucleic and amino acid residues, cannot be unambiguously assigned as DNA, RNA, or protein from the sequence, or contain residues marked as “unknown” or non‐linking residues. Individual polymer entities are classified into proteins (amino acid sequences of 30 residues or more), peptides (amino acid sequences shorter than 30 residues), DNA, or RNA.

Then, protein sequences are clustered with CD‐HIT [28] version 4.8.1 at 99% sequence identity. Peptide, RNA, and DNA sequences are clustered at 100% sequence identity. Single‐chain clustering is then aggregated to complexes, and targets that contain the same set of single‐sequence clusters are clustered together. Entries in a cluster are sorted alphabetically by PDB ID, and the first entry of a cluster is used as a representative.

To classify targets, we first search for similar polymer sequences in the PDB with BLAST+ [29] version 2.2.31. A protein sequence is marked as “easy” for a given template if it has 85% or more sequence identity to the target, at least 70% of the target sequence is covered by the template, and, for target sequences longer than 250 residues, less than 45 amino acids of the target are not covered by the target‐template alignment. A protein sequence is marked as “medium” for a given template if it has less than 85% sequence identity but an *e* value of 10^−4^ or less, and the same coverage requirements. All other protein sequences are marked as “hard.” Peptides, DNA, and RNA sequences are marked as “easy” for a given template if the sequence identity is 100% with full coverage; “hard” otherwise.

In order to derive a classification for an entry, we look for templates covering all the entities of the complex. The complex is classified as “easy” if a template can be found that covers all the individual sequences as “easy”; “medium” if the complex is not “easy,” but a template can be found that is either “easy” or “medium” for all the individual sequences; and “hard” if at least one sequence can only be mapped to a “hard” template, or if no template was found to cover the whole complex. “Easy” targets are further investigated to find targets suitable for ligand modeling challenges. If none of the “easy” templates contain the same set of ligands, the target is classified as a “ligand” target. This is then extended to every clustered entry as well.

### 3.2 Baselines

#### 3.2.1 SWISS‐MODEL and AutoDock Vina

Protein‐ligand targets are sent to a combined SWISS‐MODEL and AutoDock Vina workflow. First, the protein sequences are sent to SWISS‐MODEL [11] via API call. The top‐ranked model by Global Model Quality Estimation (GMQE) is used as a receptor for further docking. Hydrogens are added with Reduce version 4.13 [30], and the receptor is prepared with the prepare_receptor script from ADFR Suite version 1.0 [31]. Ligands are converted from SMILES with RDKit [32] version 2021.03.4; charges are removed, and protonation is added (https://github.com/jensengroup/protonator/blob/main/protonator.ipynb). An initial 3D conformation of the ligand is obtained with RDKit “EmbedMolecule,” and the ligand is further prepared with the mk_prepare_ligand.py from Meeko (https://github.com/forlilab/Meeko). AutoDock Vina version 1.2.5 [12] is run with an exhaustiveness of 32 and a box containing the entire protein receptor. No binding site detection is performed. Two flavors of AutoDock are available with the vina or the ad4 (AutoDock4) scoring functions. Pose ranking is performed by AutoDock.

#### 3.2.2 SWISS‐MODEL and Schrödinger Glide

Protein‐ligand targets are sent to a combined SWISS‐MODEL and Schrödinger Glide (Schrödinger LLC: New York, 2025) workflow. The protein receptor model is obtained as described for the SWISS‐MODEL and AutoDock Vina docking workflow. Docking is performed with the Schrodinger Suites version 2023‐3 (until September 2024) or 2024‐3 (since October 2024). The ligand SMILES are prepared with LigPrep with Epik Classic for ionization and tautomerization, a pH tolerance of 1, and up to 16 stereoisomers per ligand. The receptor is prepared with Prepwizard with the‐rehtreat option. Glide is then run with a grid spanning the entire receptor protein, and XP precision. Poses are ranked according to the PoseRank obtained from the glide output.

#### 3.2.3 AlphaFold 3

All targets containing proteins, RNA, DNA, and ligands are processed with AlphaFold 3 version 3.0.1 [15] with five seeds. PTMs are sent as CCD compound IDs. The models are ranked by “ranking_score.” As stoichiometry information is lacking during submission, complexes are assumed to contain a single copy of each chain (including ligands).

### 3.3 Evaluation

The CAMEO evaluations are performed weekly after experimental structures are released by the PDB. Only structures derived from solution NMR, X‐ray diffraction, or high resolution (≤ 4.0 Å) EM structures are used as references for the evaluation, and very large complexes (> 200 polymer chains in total, or > 100 chains of a single entity), where scoring cannot be computed in reasonable time, are excluded.

Scoring is performed by OpenStructure (version 2.9.2 at the time of writing) as described in [18]. As in CASP, models are assumed to be numbered according to the target sequence (1‐based), and deviations will result in low scores. Structure entries in the PDB can provide multiple potential biological assemblies [33], and models are initially scored against all biological assemblies of the target. Polymer scores are then reported against the assembly yielding the highest LDDT. Ligand scores are reported against the assembly resulting in the largest number of ligands scored. In case of ties, the assembly resulting in a higher sum of LDDT‐PLI or in a lower sum of BiSyRMSD is reported. LDDT‐PLI is aggregated with weighted means, with the number of heavy atoms in the ligand excluding hydrogens as the weighting factor. This prevents ions from dominating average scores. RMSD is used to calculate the fraction of successes. A ligand prediction is considered successful if the BiSyRMSD is < 2.0 Å, and the number of successes is divided by the total number of ligands in the target. Scores of the first model (Model 1) are reported on the web interface, rewarding methods that correctly assess the accuracy of their predictions. Scores for the additional models, as well as additional scores, are available in the full data downloads. A variation of polymer chain scores is provided where, after the initial scoring of the entire reference and model complexes, scores are re‐computed only on chains that were initially mapped, mitigating the penalty for missing chains for methods that are not able to predict stoichiometry. Similarly, ligand scores considering only the best prediction for each type of ligand are available, together with BiSyRMSD successes at different thresholds (1, 2, and 5 Å) and per‐ligand scores.

## 4 Conclusion

The rapid evolution of structure prediction methods, particularly those based on deep learning, has created a pressing need for continuous, unbiased benchmarking on novel data. Static datasets such as PoseBusters and Runs' N′ Poses are quickly getting incorporated into training pipelines, limiting their utility for independent evaluation. In this context, CAMEO plays a critical role by providing weekly, blind benchmarking of structure prediction methods using newly released experimental structures from the PDB. This automated framework enables developers to assess their methods in real time and supports the iterative refinement of prediction algorithms. By explicitly evaluating stoichiometry through scores penalizing missing chains and rewarding complete assemblies, CAMEO encourages the development of methods that can accurately model the full composition of macromolecular complexes.

Several open challenges in structure prediction remain, which could be tackled with data available in the PDB pre‐release. For example, benchmarking of peptide predictions, especially short ones which often contain modified residues or non‐standard amino acids, will require new prediction algorithms as well as new benchmarking methods for scoring. To better benchmark nucleic acid predictions, robust methods for assessing the difficulty of RNA and DNA will need to be developed. Scoring of post‐translational modification (PTM) predictions is implicitly captured in the LDDT score. However, the score is dominated by the large number of “standard” contacts and more sensitive scores need to be developed to capture the effects of PTMs. Finally, assessment of model quality estimates (by the prediction methods themselves as well as independent model assessment approaches) needs to be addressed. New methods and formats have been proposed in CASP15 [34] and CASP16. It remains to be seen how similar challenges can be included in CAMEO.

This manuscript reports on several detailed analyses that are not routinely performed by CAMEO, such as ligand class breakdown. We are considering ways to include such detailed analyses in the CAMEO website. The difficulty classification of targets in CAMEO is being updated regularly to reflect the current scope of modeling approaches in order to provide interesting and challenging targets to participating methods to foster further development.

Since the release of AlphaFold 2 [1] and ColabFold in 2021 [35], many state‐of‐the‐art 3D structure prediction methods have been available as standalone tools or notebooks rather than prediction servers. The AlphaFold 3 server [15] contained several usage restrictions and didn't provide an automated interface for target submission. In addition, many developers of deep‐learning prediction methods don't have the computational resources to provide servers to the public. We are evaluating options to benchmark executable workflows and notebooks instead of servers for evaluation in CAMEO.

Many successful CASP participants have leveraged CAMEO by either benchmarking their servers within the system or comparing their performance against CAMEO reference data. We encourage method developers to register their methods to the new version of CAMEO. The CAMEO platform aims to offer automated benchmarking services and training data, and to promote discussion, collaboration, and exchange among developers between the biannual CASP meetings in order to foster the development of the next generation of techniques to address the remaining challenges in structure prediction.

## Author Contributions

**Xavier Robin:** writing – original draft, data curation, conceptualization, investigation, methodology, validation, visualization, software, formal analysis, writing – review and editing. **Peter Škrinjar:** writing – original draft, data curation, investigation, methodology, validation, visualization, writing – review and editing, software, formal analysis. **Andrew M. Waterhouse:** visualization, software, writing – review and editing. **Gabriel Studer:** investigation, validation, writing – review and editing, software. **Gerardo Tauriello:** supervision, conceptualization, funding acquisition, resources, writing – review and editing, project administration. **Janani Durairaj:** supervision, investigation, writing – review and editing, software, conceptualization. **Torsten Schwede:** supervision, conceptualization, funding acquisition, resources, writing – review and editing.

## Supporting information


**Figure S1:** Protein‐ligand complex prediction comparison including RoseTTAFold All‐Atom. The success rate, defined as the percentage of ligand entities with < 2 Å RMSD, across five servers for (A) all ligand predictions, (B) across different ligand categories, and (C) across different ligand similarity bins for the ligands in the drug‐like category. (D) The distribution of LDDT‐PLI values for five servers across different ligand similarity bins for the ligands in the drug‐like category. Servers: AlphaFold 3 (AF3, blue), RosettaFoldAllAtom (RFAA, cyan), SWISS‐MODEL homology modeling and ligand docking with Schrödinger Glide (SM_Glide, orange), Autodock Vina with vina scoring (SM_Vina_vina, green) and autodock4 scoring (SM_Vina_ad4, red). There are 385 targets in the common subset with RoseTTAFold All‐Atom, which results in 854 ligand entities.

## Data Availability

The data that support the findings of this study are available from the corresponding author upon reasonable request.
