# Supplementary figures and images for: Beyond Single Chains: Benchmarking Macromolecular Complex Prediction Methods With the Continuous Automated Model EvaluatiOn (CAMEO)
Source: Proteins. 2025 Sep 28;94(1):403–13. doi: 10.1002/prot.70060 (PMC12750032; doi:10.1002/prot.70060)

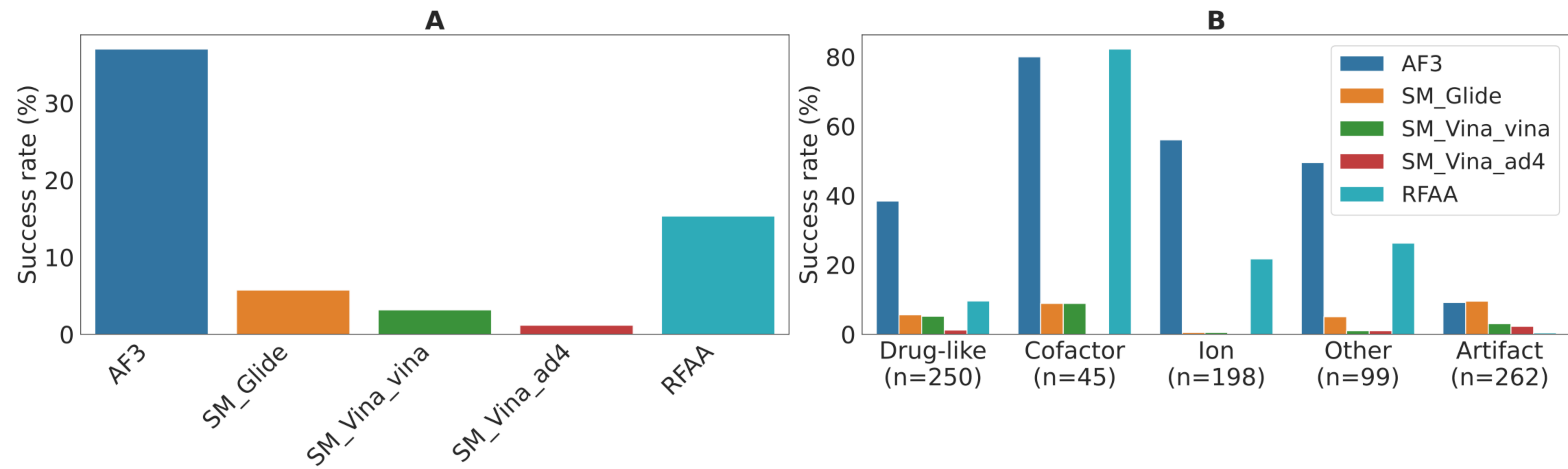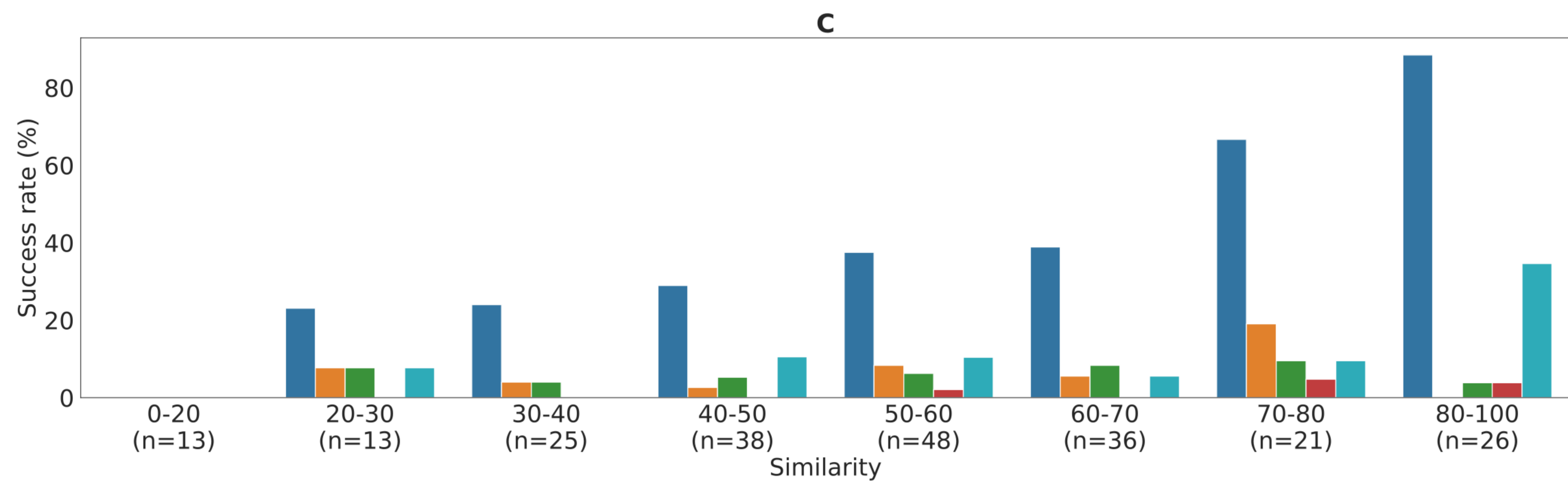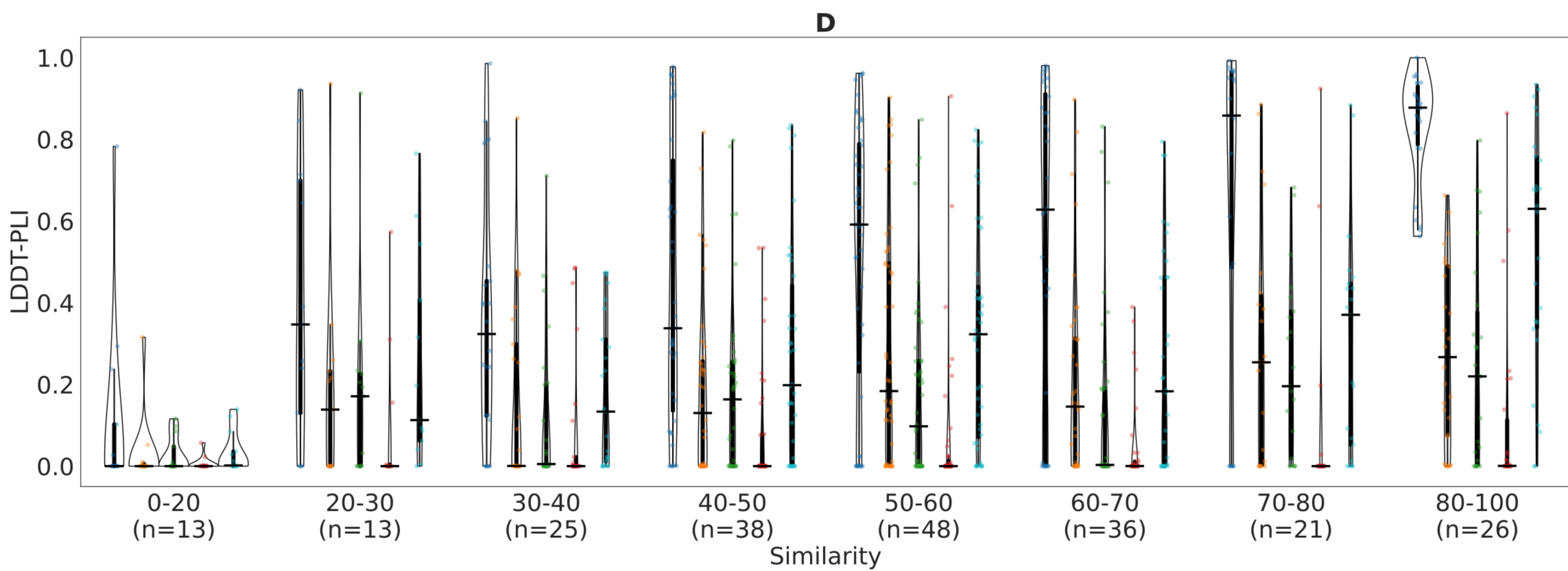

Supplement: Supplementary file 1 — Figure S1: Protein‐ligand complex prediction comparison including RoseTTAFold All‐Atom. The success rate, defined as the percentage of ligand entities with < 2 Å RMSD, across five servers for (A) all ligand predictions, (B) across different ligand categories, and (C) across different ligand similarity bins for the ligands in the drug‐like category. (D) The distribution of LDDT‐PLI values for five servers across different ligand similarity bins for the ligands in the drug‐like category. Servers: AlphaFold 3 (AF3, blue), RosettaFoldAllAtom (RFAA, cyan), SWISS‐MODEL homology modeling and ligand docking with Schrödinger Glide (SM_Glide, orange), Autodock Vina with vina scoring (SM_Vina_vina, green) and autodock4 scoring (SM_Vina_ad4, red). There are 385 targets in the common subset with RoseTTAFold All‐Atom, which results in 854 ligand entities. [file PROT-94-403-s001.pdf]
